# Maternal Attachment Style, Interpersonal Trauma History, and Childbirth-Related Post-traumatic Stress

**DOI:** 10.3389/fpsyg.2018.02379

**Published:** 2018-11-28

**Authors:** Anna L. MacKinnon, Sarah Houazene, Stephanie Robins, Nancy Feeley, Phyllis Zelkowitz

**Affiliations:** ^1^Department of Psychiatry, Jewish General Hospital, Montreal, QC, Canada; ^2^Lady Davis Institute for Medical Research, Montreal, QC, Canada; ^3^Department of Psychology, McGill University, Montreal, QC, Canada; ^4^Department of Psychology, University of Montreal, Montreal, QC, Canada; ^5^Centre for Nursing Research, Jewish General Hospital, Montreal, QC, Canada; ^6^Ingram School of Nursing, McGill University, Montreal, QC, Canada; ^7^Department of Psychiatry, McGill University, Montreal, QC, Canada

**Keywords:** childbirth, post-traumatic stress, attachment, trauma, PTSD, postpartum

## Abstract

Childbirth-related post-traumatic stress has potentially negative and enduring consequences for the well-being of women and their families. Although research to date has identified attachment style and trauma history as individual risk factors, they have yet to be examined as integrative processes in the development and maintenance of childbirth-related post-traumatic stress. The current investigation aimed to examine whether attachment style may moderate the impact of a history of interpersonal trauma on initial levels and the rate of change in post-traumatic stress symptomatology across the first 6 months of the postpartum period. A large community sample of women were recruited from two Canadian urban hospitals. Childbirth-related post-traumatic stress symptoms were assessed longitudinally at 5 weeks, 2 months, and 6 months postpartum. Latent growth curve modeling (*n* = 251) revealed that attachment style moderated the impact of a history of interpersonal trauma on initial levels and the rate of change in post-traumatic stress symptomatology, while controlling for other well-established psychosocial (e.g., trait anxiety, previous psychopathology, lack of perceived support) and childbirth-related (e.g., mode of birth, labor pain, subjective experience) risk factors. More secure attachment conferred resiliency and more fearful attachment conferred vulnerability among women without a history of interpersonal trauma, while more preoccupied and more dismissing attachment conferred resiliency among women with a history of interpersonal trauma. These findings highlight the importance of understanding the integrative processes among risk and protective factors underlying the development of and ability to cope with childbirth-related post-traumatic stress. Attachment style and trauma history, which can be quickly measured, should be considered as targets in antenatal screening.

## Introduction

Although childbirth is generally expected to be a time of joy, there is increasing evidence from research and clinical practice that many women experience distress and perceive childbirth to be a traumatic event (for reviews see Ayers, 2004; Grekin and O’Hara, 2014; Dekel et al., 2017). Indeed, childbirth-related post-traumatic stress among women is a concerning and often overlooked mental health problem. The diagnosis of postpartum post-traumatic stress disorder (PTSD) has a reported average prevalence of 3.1% in community samples and 15.7% in at-risk samples such as women with a history of trauma, previous psychopathology, or perinatal complications (Grekin and O’Hara, 2014). In addition, estimates of the prevalence of postpartum PTSD symptomatology range from 5.5 to 30.1% (e.g., Soet et al., 2003; White et al., 2006). Childbirth-related post-traumatic stress has not only immediate but also long-term effects on women’s well-being and romantic relationships (e.g., Ayers et al., 2006), mother-infant attachment (e.g., Davies et al., 2008), and possibly children’s neurodevelopment (e.g., Koen et al., 2017). Given that childbirth is a foreseeable event, it is imperative to improve our understanding of the risk factors for experiencing it as traumatic.

A growing body of research has identified factors implicated in the development of postpartum PTSD. Psychosocial risk factors, which often pre-date childbirth, include high trait anxiety (e.g., Soet et al., 2003), previous psychopathology and stressful or traumatic life events (e.g., Ayers et al., 2016), as well as a history of sexual abuse (e.g., Wosu et al., 2015). Childbirth-related risk factors, which arise during labor and birth, include a subjective experience of childbirth as unsatisfactory or painful (e.g., Dekel et al., 2017) and emergency care or invasive obstetric intervention such as the use of forceps or vacuum (e.g., Rowlands and Redshaw, 2012). Similarly, a recent study by our team demonstrated that having low-birth weight infants who require neonatal intensive care unit (NICU) hospitalization placed women at higher risk for experiencing childbirth-related PTSD symptoms (Feeley et al., 2017).

In terms of psychological factors, examination of attachment style may shed light on the individual differences found in experiencing childbirth-related post-traumatic stress. In the general population, secure attachment has been associated with less PTSD symptomatology, while insecure attachment, including fearful, preoccupied and dismissing styles, has been associated with more symptoms and an increased risk of a diagnosis of PTSD (e.g., O’Connor and Elklit, 2008; Woodhouse et al., 2015). This pattern of resiliency versus vulnerability may reflect the impact of attachment style on psychological functioning, which according to attachment theory involves the programming of internal working models or mental representations about the self, others, and the perceived safety of the external world (Bowlby, 1969). With a positive sense of self and others, individuals with a secure attachment style are likely more comfortable seeking support when needed and thus may be better able to adapt to adverse events (Fraley et al., 2006; O’Connor and Elklit, 2008). In contrast, insecure attachment may interfere with effective coping and make it more difficult to regulate distress (Ein-Dor et al., 2010).

Little research has investigated attachment style in relation to childbirth-related post-traumatic stress. One study of couples demonstrated that higher levels of attachment anxiety and avoidance were related to more symptoms of post-traumatic stress at 6 weeks and 3 months postpartum among women and their male partners (Iles et al., 2011). In a second study (Ayers et al., 2014), avoidant attachment style was associated with PTSD symptoms at 3 months postpartum, particularly for women who had operative births (assisted vaginal or cesarean section). The role of attachment style in the long-term maintenance of childbirth-related post-traumatic stress has yet to be investigated beyond 3 months postpartum. Because longitudinal studies have revealed that PTSD can present at 6 months postpartum (e.g., Zaers et al., 2008) in line with the American Psychiatric Association criteria for “delayed expression” (American Psychiatric Association, 2013), studies with longer time frames are warranted. Moreover, given the possible negative effects on mental health and parenting (McDonald et al., 2011) following women longer into the postpartum period and examining attachment style may help to identify individuals whose symptoms of post-traumatic stress decline more slowly, resulting in more detrimental consequences for emotional and social well-being, and parenting.

Further, in the last decade focus has shifted from the identification of individual risk factors toward elucidating the integrative processes that convey risk for developing PTSD. The comprehensive social ecological framework put forward by Charuvastra and Cloitre (2008) posits that vulnerability for experiencing post-traumatic stress is largely dependent upon social processes, namely interpersonal trauma and attachment style. More specifically, a history of childhood abuse or early life adversity may contribute to the development of PTSD in adulthood, such that these individuals develop enduring insecure attachment in close relationships, reduced expectations of social support, and an inability to regulate emotions in times of distress (Charuvastra and Cloitre, 2008). In line with this framework, recent evidence suggests that attachment style moderates the impact of interpersonal trauma on post-traumatic stress. For example, secure attachment was found to buffer the effects of childhood abuse history and genetic risk alleles on the severity of PTSD symptoms among men and women who served in the military (Tamman et al., 2017). A history of physical abuse and sexual victimization was associated with more post-traumatic stress symptomatology for women with higher dismissing attachment (Sandberg, 2010). Such models have yet to be explored for childbirth-related post-traumatic stress, but are of particular importance since the physical requirements and role transitions that accompany childbirth may increase the salience of trauma- and attachment-related issues (Leeners et al., 2006; Scheidt et al., 2012; Choi and Sikkema, 2016).

The current study aimed to build on and extend existing research by examining the integrative processes of attachment style and interpersonal trauma in the development and longer-term maintenance of childbirth-related post-traumatic stress symptomatology among a large community sample of women. Secondary analyses of the data from our team’s recent study (Feeley et al., 2017) provided the unique opportunity to explore the differential vulnerability conveyed by attachment styles on childbirth-related PTSD symptoms as assessed at 5 weeks, 2 months, and 6 months postpartum. Latent growth curve modeling was utilized to test whether attachment style moderates the impact of a history of interpersonal trauma on initial levels and rate of change in post-traumatic stress symptomatology, while controlling for other well-established psychosocial (e.g., trait anxiety, previous psychopathology, lack of perceived support, recent stressful life events) and childbirth-related (e.g., mode of delivery, labor pain, subjective experience) risk factors (Soet et al., 2003; Rowlands and Redshaw, 2012; Wosu et al., 2015; Ayers et al., 2016; Dekel et al., 2017). Given secure attachment may confer resiliency in response to stressful events (Woodhouse et al., 2015), such as childbirth, we expected that higher secure attachment ratings would predict experiencing fewer symptoms of PTSD at 5 weeks postpartum and a larger decrease in symptom severity over time, among women with and without a history of interpersonal trauma. Whereas insecure attachment may impede coping and regulation of distress (Ein-Dor et al., 2010), we expected that insecure attachment ratings would predict higher initial levels of PTSD symptoms at 5 weeks postpartum and a smaller reduction in symptom severity over time, particularly among women with a history of interpersonal trauma, as they are more vulnerable to experiencing post-traumatic stress (Goodman et al., 2016).

## Materials and Methods

The current investigation is a part of a larger longitudinal study designed to explore the development of PTSD symptomatology in a community sample of women; the methodology has been described elsewhere (Feeley et al., 2017).

### Participant Recruitment

Women were approached within 24–48 h post-birth from the postpartum unit of an urban teaching hospital in Montreal, Canada or 1-week post-birth from the NICU of this same hospital as well as the NICU of a nearby pediatric hospital, if their infant was in stable medical condition. Women were eligible to participate if they were proficient in French or English, had birthed a live singleton baby and lived close enough to the hospital (80 km) for home visit assessments. A total of 298 women were recruited into one of four birth groups: 94 with vaginal births; 59 with a vaginal birth and low birth weight requiring NICU admission; 62 with planned cesarean births; and 83 with emergency cesarean births. Inclusion criteria for each birth group were previously reported (see Feeley et al., 2017 for details). Recruitment took place between March 2011 and April 2013.

### Measures

#### Background Information

Participants were asked to provide socio-demographic information such as their age, marital status, level of education, income, immigrant status, number of years living in Canada, and main language spoken. Medical records were reviewed for information on parity, gestational age, and sex of child.

#### Childbirth-Related Risk Factors

Dichotomous variables for membership (*yes/no*) to each birth group were created to represent mode of delivery (vaginal, vaginal with a neonatal intensive care admission, planned cesarean section, and emergency cesarean section). Women were asked to rate the level of pain experienced during labor by responding to the question: “How painful were your labor and birth?” on a scale from 0 (*No pain*) to 10 (*Maximum imaginable*). This scale is reliable in its assessment of acute pain (Breivik and Stubhaug, 2008). Women also rated their subjective experience of childbirth by responding to one item from the Canadian Maternity Experiences Survey (Bartholomew and Public Health Agency of Canada, 2009): “Overall, would you describe your experience of labor and birth as...?” Responses were rated on a four-point scale from -2 (*very negative*) to +2 (*very positive*).

#### Psychosocial Risk Factors

The 12-item self-report Postnatal Risk Questionnaire (PNRQ; Austin and Priest, 2005) taps a range of well-established psychosocial risk factors including trait anxiety, previous psychopathology, lack of perceived support, and recent stressful life events that are associated with postpartum psychopathology. The PNRQ includes both yes/no and Likert scale rated items, where higher scores indicate a greater level of risk. The PNRQ is based on the well-validated Antenatal Risk Questionnaire (ANRQ; Austin et al., 2013) and has demonstrated high acceptability for use among women during the first year after childbirth (Christl et al., 2013). For the purposes of the current investigation, three items were removed from the PNRQ (see below), and, without these, the total score can range from 6 to 67.

#### Interpersonal Trauma History

Three items were removed from the PNRQ to compute a dichotomous variable for interpersonal trauma history (Christl et al., 2015). A woman was deemed to have a history of interpersonal trauma if she endorsed any of the following: perceiving her mother not to be emotionally supportive during childhood (score > 4), experiencing emotional abuse during childhood, or experiencing sexual or physical abuse during her lifetime.

#### Attachment Style

Adult attachment style was assessed using the self-report Relationship Questionnaire (RQ; Bartholomew and Horowitz, 1991), which comprises both a categorical component and continuous ratings. Respondents are first presented with four short paragraphs that each describe an attachment style and are asked to indicate which one best defines their close relationships, these include: Secure (*It is easy for me to become emotionally close to others. I am comfortable depending on others and having others depend on me. I don’t worry about being alone or having others not accept me*), Fearful (*I am uncomfortable getting close to others. I want emotionally close relationships, but I find it difficult to trust others completely, or to depend on them. I worry that I will be hurt if I allow myself to become too close to others*), Preoccupied (*I want to be completely emotionally intimate with others, but I often find that others are reluctant to get as close as I would like. I am uncomfortable being without close relationships, but I sometimes worry that others don’t value me as much as I value them*) and Dismissing (*I am comfortable without close personal relationships. It is very important to me to feel independent and self-sufficient, and I prefer not to depend on others or have others depend on me*). Then respondents are asked to rate to what extent each description relates to them on a scale from 1 (*not at all like me*) to 7 (*very much like me*). The RQ demonstrates convergent construct validity with interview measures of attachment style (Bartholomew and Horowitz, 1991). Only the continuous ratings were used in the current investigation, as attachment is now considered to fall along a continuum of each style (Fraley et al., 2000). When the secure item is reversed to score it in the same way as the insecure items, adequate internal consistency reliability was established (α = 0.60) among the four continuous ratings.

#### Childbirth-Related Post-traumatic Stress

Postpartum symptoms of childbirth-related post-traumatic stress were assessed using the revised Perinatal PTSD Questionnaire (PPQ; Callahan et al., 2006). This 14-item self-report measure captures intrusive thoughts, avoidance of stimuli and hyperarousal associated with childbirth. Women responded to questions that reflect experiences they may have had since childbirth for the first assessment, and since the preceding data collection time point for all subsequent assessments. Items are rated on a five-point Likert scale from 0 (*not at all*) to 4 (*often, for more than 2 weeks*). Total scores can range from 0 to 56, with a clinical cut-off score of 19 or higher indicating a level of distress that warrants a referral to a mental health professional. The revised PPQ has high internal consistency (α = 0.80 to 0.90), very good test–retest reliability (*r* = 0.92) and good convergent and divergent construct validity (Callahan et al., 2006). In the current study, satisfactory internal consistency (α = 0.77 to 0.84) and test–retest (*r* = 0.68 to 0.73) reliability were observed.

### Procedures

Participants were assessed at four different time points: in person at enrolment (T1), at 5 weeks postpartum via a phone call (T2), and in person at 2 months (T3) and 6 months (T4) postpartum during home visits. Participants were asked to provide background information and complete the childbirth-related risk items at T1. The PNRQ was administered at T3 and the PPQ was administered at T2, T3, and T4. The RQ was administered at T4. Chart review was performed immediately following recruitment to confirm eligibility between the planned and emergency cesarean section groups.

### Ethics Statement

Research ethics boards of the hospitals where recruitment took place approved the study protocol. Women who agreed to participate provided written informed consent, in accordance with the Declaration of Helsinki.

### Data Analyses

Descriptive statistics were computed using SPSS Statistics 25 (IBM, United States). Latent growth curve modeling was conducting using LISREL 8.8 (SSI, United States). Two latent factors were estimated: an intercept representing the average level of PTSD symptomatology at baseline (i.e., 5 weeks postpartum) and a slope representing the linear rate of change in PTSD symptomatology over time (e.g., from 5 weeks to 2 months to 6 months postpartum). The continuous scales for each attachment style and the dichotomous variable for a history of interpersonal trauma were used as predictors of the intercept and slope. We controlled for mode of delivery, labor pain, subjective experience of childbirth, and psychosocial risk (i.e., PNRQ without the three trauma items) by including them as predictors of the intercept. Given unequal intervals, time was modeled as the number of months from preceding measurement points (i.e., 0 for 5 weeks, 1 for 2 months, and 4 for 6 months postpartum). Acceptable model fit is indicated by a non-significant chi-square, a root mean square error of approximation (RMSEA) smaller than 0.06, a standardized root mean square residual (SRMR) smaller than 0.08, as well as a comparative fit index (CFI), a normed fit index (NFI) and a non-normed fit index (NNFI) all larger than 0.95 (Jöreskog and Sörbom, 1986; Schermelleh-Engel et al., 2003). Overall, 7.67% of the data was missing, which was handled using listwise deletion.

## Results

### Participant Characteristics

Of the 298 women who participated, 164 (55.0%) were not born in Canada, 288 (96.6%) reported being married or living with a partner, 166 (55.7%) were first-time mothers, and 152 (48.1%) gave birth to male infants. On average, women were 31.86 (*SD* = 5.26) years of age and had 15.86 (*SD* = 3.36) years of education. Participants who dropped out (*n* = 41) did not differ from those who completed the study (*n* = 257) on any background variables other than years living in Canada; those who completed the study had lived in Canada significantly longer (*t* = -2.059, *p* = 0.04). Dropouts also did not differ from those retained on ratings of labor pain or childbirth experience at T1, PPQ scores at T2 or T3, nor on the PNRQ total or interpersonal trauma scores at T3. First-time mothers did not significantly differ from other women on any of the main study variables including trauma history, attachment ratings, or PTSD symptoms.

### Descriptive Statistics

Descriptive statistics for the continuous variables are presented in Table 1. Of the 275 women who completed the PNRQ, the responses of 47 (17.1%) indicated a history of interpersonal trauma. Attachment style ratings fell within one standard deviation of findings in other community samples of women (e.g., Sandberg, 2010). Baseline levels of post-traumatic stress symptoms fell within one standard deviation of previous reports in healthy and at-risk samples using the PPQ (e.g., Kim et al., 2015). As previously reported (Feeley et al., 2017), the number of participants who scored above the clinical cut-off for the PPQ at each time point were 33 (11.1%) at 5 weeks postpartum, 24 (8.6%) at 2 months postpartum, and 16 (6.2%) at 6 months postpartum.

**Table 1:** Descriptive statistics for continuous study variables.

	Full sample			Trauma history			No trauma		
Variable	*M*	*SD*	*Range*	*M*	*SD*	*Range*	*M*	*SD*	*Range*
Psychosocial risk	20.92	10.18	7–49	25.72	10.35	10–49	19.93	9.88	7–48
Childbirth-related risk									
Labor pain	7.18	3.64	0–10	7.43	3.79	0–10	7.18	3.61	0–10
Subjective experience	3.82	1.03	1–5	3.77	1.09	1–5	3.82	1.03	1–5
Attachment ratings									
Secure	5.17	1.77	1–7	4.74	2.03	1–7	5.26	1.70	1–7
Fearful	2.48	1.69	1–7	2.90	1.79	1–7	2.40	1.66	1–7
Preoccupied	2.28	1.63	1–7	2.62	1.86	1–7	2.21	1.57	1–7
Dismissive	2.74	1.88	1–7	3.21	2.01	1–7	2.65	1.85	1–7
PTSD symptoms									
5 weeks postpartum	8.59	6.97	0–37	12.07	8.57	0–29	7.64	5.91	0–24
2 months postpartum	7.38	7.09	0–34	11.07	8.10	0–34	6.70	6.68	0–34
6 months postpartum	6.46	6.32	0–31	10.10	7.63	0–28	5.75	5.80	0–31

*Psychosocial risk factors were measured using the Postnatal Risk Questionnaire (PNRQ) total without the three trauma items.*

### Latent Growth Curve Models

**FIGURE 1:**
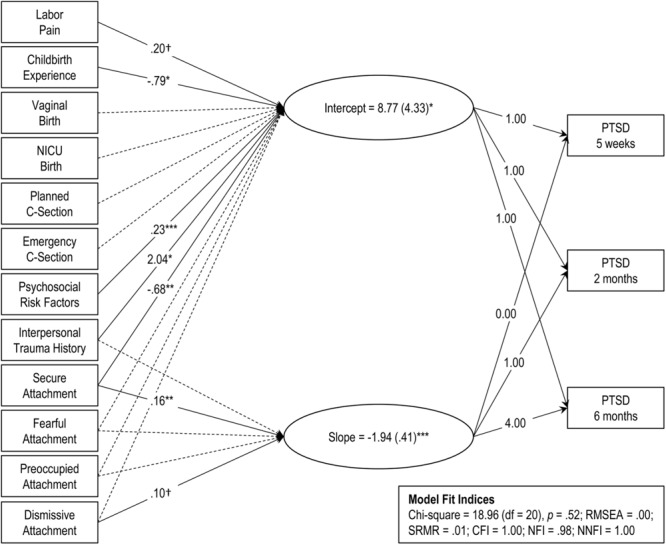
Latent growth curve model of initial PTSD symptoms (intercept) and rate of change over time (slope) for the full sample (*n* = 251). Model fit indices are presented in the inset. Rectangles represent manifest (measured) variables and ovals represent latent factors. Dashed lines represent non-significant coefficients (*p* > 0.10). Solid lines represent statistically significant coefficients and are presented with unstandardized coefficient values. Significance levels indicated by: †*p* < 0.10, ^∗^*p* < 0.05, ^∗∗^*p* < 0.01, and ^∗∗∗^*p* < 0.001.

Listwise deletion removed 47 women as they did not have information on one or more of the variables included in the model. Then, a linear latent growth curve model for the full sample of the remaining women with and without a history of trauma (*n* = 251) was estimated (see Figure 1). According to the indices, model fit was satisfactory. The significant positive intercept indicates a moderate level of PTSD symptoms at 5 weeks postpartum and the significant negative slope indicates a decline in PTSD symptoms over time. More psychosocial risk factors (e.g., trait anxiety, previous psychopathology, lack of perceived support), greater labor pain, negative childbirth experiences, lower secure attachment, and a history of interpersonal trauma all predicted a higher baseline (i.e., higher intercept). Higher secure and dismissing attachment ratings predicted a greater decline (i.e., steeper slope). Mode of delivery was not related to the intercept or slope, and therefore was not included in subsequent analyses.

**FIGURE 2:**
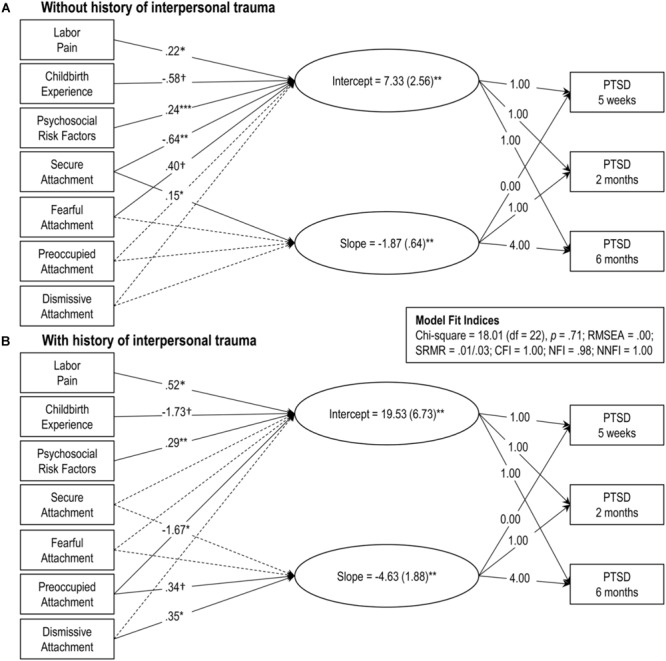
Linear latent growth curve models for women with (**B**, *n* = 40) and without (**A**, *n* = 211) a history of interpersonal trauma. Model fit indices are presented in the inset. Rectangles represent manifest (measured) variables and ovals represent latent factors. Dashed lines represent non-significant coefficients (*p* > 0.10). Solid lines represent statistically significant coefficients and are presented with unstandardized coefficient values. Significance levels indicated by: †*p* < 0.10, ^∗^*p* < 0.05, ^∗∗^*p* < 0.01, ^∗∗∗^*p* < 0.001.

**FIGURE 3:**
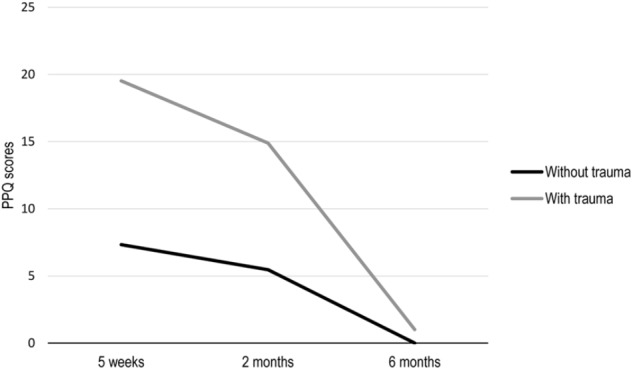
Graphical representation of the estimated mean scores of childbirth-related PTSD symptoms at each time point (i.e., time^∗^slope) from the linear latent growth curve models for women with (*n* = 40) and without (*n* = 211) a history of interpersonal trauma. PPQ = Perinatal PTSD Questionnaire.

Second, to test whether attachment style moderates the impact of interpersonal trauma on postpartum PTSD symptoms, a multiple sample analysis was conducted estimating linear latent growth curve models for women with (*n* = 40) and without (*n* = 211) a history of interpersonal trauma using the attachment ratings as predictors while controlling for the psychosocial and childbirth-related risk factors (see Figures 2A,B). Overall fit was satisfactory. As can be seen in Figures 2 and 3, the intercept was higher and the slope steeper for women with a history of interpersonal trauma, though post-traumatic stress symptoms declined over time among both groups. Among women without a history of interpersonal trauma, higher secure attachment ratings predicted a lower baseline (i.e., lower intercept) and a greater decline (i.e., steeper slope) for PTSD symptoms, whereas higher fearful attachment ratings predicted a higher baseline (i.e., higher intercept) but not slope. Among women with a history of interpersonal trauma, higher preoccupied attachment ratings predicted a lower baseline (i.e., lower intercept), while higher preoccupied and dismissing attachment ratings both predicted a greater decline (i.e., steeper slope).

## Discussion

To our knowledge, this study is the first to demonstrate in a large community sample of women that maternal attachment style moderates the impact of a history of interpersonal trauma on the development and maintenance of childbirth-related post-traumatic stress symptomatology 6 months into the postpartum period. Latent growth curve modeling revealed a differential pattern of associations between attachment style ratings with initial PTSD symptoms and change over time, such that secure attachment conferred resiliency and fearful attachment conferred vulnerability among women without a history of interpersonal trauma. Contrary to our expectations, preoccupied, and dismissing attachment both conferred resiliency among women with a history of interpersonal trauma.

Regardless of attachment style, having a history of interpersonal trauma may increase women’s vulnerability to experiencing childbirth as traumatic, as indicated by the average level of PTSD symptoms (i.e., intercept) above the clinical cut-off at 5 weeks postpartum observed in this study. This is in line with a recent review (Choi and Sikkema, 2016), suggesting that a history of childhood maltreatment is one of the strongest predictors of perinatal mood and anxiety disorders, including PTSD. Despite higher initial levels, these women have a larger decrease in PTSD symptoms over time, which may reflect previous experience dealing with trauma. Nevertheless, the differential pattern of findings observed between women with and without a history of trauma confirms that attachment style moderates the impact of trauma history and supports the social ecological framework (Charuvastra and Cloitre, 2008) proposition that integrative social processes underlie the development of post-traumatic stress.

Among women without a history of interpersonal trauma, more secure attachment was associated with fewer PTSD symptoms following childbirth and a larger decrease over time. This is consistent with previous research (e.g., Woodhouse et al., 2015) suggesting that secure attachment confers resiliency in response to stressful life events. In line with attachment theory, women with more secure attachment have likely formed trusting relationships and sought social support, thus better equipping them to cope with the challenges often involved in childbirth and parenting a new infant. In terms of insecure attachment, more fearful attachment predicted higher initial levels of PTSD symptoms among women without a history of trauma. These findings parallel previous studies indicating that more avoidant attachment, which encompasses fearful attachment, is associated with childbirth-related symptoms of PTSD during the postpartum period (Iles et al., 2011; Ayers et al., 2014). Since childbirth often involves relying on others (e.g., healthcare providers, family members, partners), women with the negative view of self (as unworthy of care) and others (as untrustworthy) characteristic of fearful attachment may have difficulty with obtaining support or feel a lack of control, which in turn may render them more vulnerable to experiencing childbirth as traumatic (e.g., Soet et al., 2003). In terms of underlying biological mechanisms, individuals with fearful attachment may have heightened hypothalamic-pituitary-adrenal (HPA) axis reactivity given their increased tendency to perceive the external world as unsafe and be hypervigilant to threat. Indeed, a recent study observed that women with a fearful attachment style did not exhibit the cortisol profile or attenuation of endocrine response that is expected during pregnancy, whereas women with secure attachment did (Costa-Martins et al., 2016).

Among women with a history of interpersonal trauma, secure attachment was not observed to be protective, rather more preoccupied and dismissing attachment were. This is consistent with previous research demonstrating it was only among women *without* a history of childhood sexual abuse that higher secure attachment ratings predicted less trauma symptoms (Aspelmeier et al., 2007). Although preoccupied attachment has been associated with more post-traumatic stress symptomatology among adults with a history of childhood abuse (Muller et al., 2000) here it was associated with fewer symptoms following childbirth and a larger decline over the postpartum period. Given their desire to be close with others, childbirth may not be experienced as traumatic for women with more preoccupied attachment as it is typically a time of increased social support from others and provides a new relationship with an infant who will be dependent on them regardless of their negative view of self. Correspondingly, Scheidt et al. (2012) observed that women with preoccupied attachment experience more post-traumatic stress symptoms after perinatal loss (e.g., miscarriage, stillbirth), which may reflect distress about no longer having an infant who would meet their attachment needs. In contrast to previous findings (e.g., Sandberg, 2010) more dismissing attachment was related to a larger decline in post-traumatic stress symptoms among women with a history of interpersonal trauma. It has been suggested that individuals with dismissing attachment may be less prone to experiencing PTSD in response to stressful events because they have a positive view of the self (as independent) and a tendency to distance themselves from or suppress negative emotions (Muller et al., 2001; Declercq and Palmans, 2006).

The current investigation extends previous research on childbirth-related post-traumatic stress beyond the identification of individual risk factors by examining the integrative processes of attachment and interpersonal trauma history in a large community sample of women. Further, we utilized latent growth curve modeling, an advanced statistical approach, to track the initial levels and change in symptoms longitudinally from 5 weeks to 6 months postpartum. However, the findings may be limited by the study design of measuring attachment at 6 months postpartum. Although it has been suggested that attachment style can change after stressful life events (Davila and Cobb, 2004) and the transition to parenthood in particular (Bowlby, 1988; Simpson et al., 2003), research findings are inconsistent and recent longitudinal evidence demonstrated that parent attachment orientation remained stable following childbirth (Galdiolo and Roskam, 2017). Similarly, the measurement of cumulative psychosocial risk and interpersonal trauma history at 2 months postpartum limited the ability to capture and control for subsequent stressors that may have impacted symptomatology at 6 months. Future research could include prenatal measurement in order to take into account symptoms of psychopathology occurring during pregnancy and isolate risk factors for experiencing childbirth as traumatic (Ayers and Pickering, 2001). Although well-validated and cost-efficient instruments were utilized, the current study was limited by the exclusive use of self-report measures. Future studies with more comprehensive clinician- or researcher- administered assessments, such as the Adult Attachment Projective (George and West, 2001), may provide a more robust test of our model. Although it has been suggested that first-time mothers may be more likely to experience childbirth-related distress (e.g., Wijma et al., 1997; Parfitt and Ayers, 2014), accumulating evidence from recent reviews and meta-analyses indicates the effect of parity is inconsistent (e.g., Andersen et al., 2012; Ayers et al., 2016) and may be outweighed by mode of delivery (Soderquist et al., 2002). The current study included mode of delivery as a control variable and first-time mothers did not significantly differ from other women on any of the main study variables including trauma history, attachment ratings, or PTSD symptoms. Finally, interpretation and generalization of the findings should be made with caution given the use of a community sample.

The findings of the current study have important implications for screening and targets for prevention as they help to better identify women vulnerable to experiencing childbirth-related post-traumatic stress, which in turn can negatively impact infant attachment and development (e.g., Davies et al., 2008; Koen et al., 2017) although the mechanisms through which this occurs remain unclear. Interestingly, postpartum PTSD symptoms were not related to maternal parenting behavior in this sample (Feeley et al., 2017), however, results from recent studies indicate that postpartum PTSD may interfere with a mother’s emotional bonding to her infant (Muzik et al., 2016; Dekel et al., 2018). Future research is needed to investigate whether the differential pattern of susceptibility for childbirth-related post-traumatic stress observed in the current study also leads to different effects on the mother-infant relationship and in turn child development.

At 5 weeks postpartum, this study identified a small but significant number of women who were likely suffering from levels of post-traumatic stress that would require referral to a mental health care provider. Unlike the focus on depression, childbirth-related post-traumatic stress has yet to be part of routine screening or conversation in perinatal healthcare. Currently, the Healthy Pregnancy – Canada website highlights that 1 in 10 women may suffer from depression (Emotional Health, 2012), but there is no mention of PTSD. For those whose post-traumatic stress symptoms persist, there is some evidence that their partner also suffers from symptoms (Iles et al., 2011), and work on PTSD in other contexts has shown that social support begins to deteriorate post-trauma (Kaniasty and Norris, 1993) which may lead to further isolation. While some might argue there is room for post-traumatic growth after childbirth, recent work by Sawyer et al. (2012) revealed that rates of post-traumatic growth in postpartum women were low. Childbirth-related post-traumatic stress clearly warrants more clinical attention. Indeed, Vesel and Nickasch (2015) proposed a new model for the prevention and treatment of childbirth-related post-traumatic stress. Briefly, prevention would involve the training of maternal care staff to screen and identify women for prenatal vulnerabilities using standardized measures. Health care providers could further mitigate distress by acknowledging patient concerns through open and collaborative communication, helping prepare women for birth with realistic expectations, and by ensuring that there is continuous care. Ongoing support provided by a doula or midwife throughout the prenatal period and into postpartum was recommended, as has been suggested previously (Hatem et al., 2008). The authors also recommended that any women who report experiencing labor and birth as traumatic, be debriefed and referred to mental health services as needed. Evidenced-based treatments for PTSD (e.g., trauma-focused cognitive behavioral therapy) that have shown promise in the context of childbirth were endorsed. These guidelines are not without inherent challenges: women may not view a midwife or other social support as a necessary component of maternity care, those with symptoms of anxiety may not consider themselves to be suffering from a mental health problem and thus may find a referral to a mental health care provider stigmatizing (O’Mahen and Flynn, 2008). Women in the perinatal period and those seeking mental health services for PTSD have reported numerous barriers to accessing treatment such as expense, inability to obtain an appointment, transportation, childcare, and availability of qualified clinicians (e.g., Dennis and Chung-Lee, 2006; Thornicroft et al., 2018). Further research is needed to test and develop such pathway models in order to better care for the health of women and their families.

## Conclusion

The findings of the current investigation highlight the importance of understanding the integrative processes that contribute to the development and maintenance of childbirth-related post-traumatic stress. Fearful attachment may only confer vulnerability among women without a history of interpersonal trauma and secure attachment may not confer resiliency among those with a history of interpersonal trauma. Given that childbirth is foreseeable and potentially stressful life event, both attachment style and trauma history can be simply and quickly assessed as part of prenatal screening, providing earlier opportunities to mobilize support and increase a sense of safety for women identified as at-risk, through trauma informed care.

## Author Contributions

NF and PZ designed and wrote the protocol. SH managed the literature searches and wrote the initial draft of the manuscript. AM conducted and interpreted some of the statistical analyses, prepared all tables and figures, and edited the manuscript. SR co-supervised the data collection, analyses, and writing. All authors contributed to and have approved the final manuscript.

## Conflict of Interest Statement

The authors declare that the research was conducted in the absence of any commercial or financial relationships that could be construed as a potential conflict of interest.
